# A positive attitude among primary healthcare providers predicts better hepatitis B prevention practices: evidence from a cross-sectional survey in Wakiso district, Central Uganda

**DOI:** 10.1080/21642850.2021.1904935

**Published:** 2021-04-07

**Authors:** Tonny Ssekamatte, John Bosco Isunju, Paul Alex Kimoga Zirimala, Samuel Etajak, Saul Kamukama, Mathias Seviiri, Mary Nakafeero, Aisha Nalugya, Solomon Tsebeni Wafula, Edwinah Atusingwize, Justine. N. Bukenya, Richard K. Mugambe

**Affiliations:** a Department of Disease Control and Environmental Health, Makerere University School of Public Health, Kampala, Uganda; b Department of Health, Wakiso District Local Government, Wakiso, Uganda; c Department of Community Health and Behavioural Sciences, Makerere University School of Public Health, Kampala, Uganda; d Statistical Genetics, QIMR Berghofer Medical Research Institute, Brisbane, Australia; e Department of Epidemiology and Biostatistics, Makerere University School of Public Health, Kampala, Uganda

**Keywords:** Hepatitis B, knowledge, attitude, practice

## Abstract

**Background:** Hepatitis B Virus (HBV) infection is an important occupational health risk among primary healthcare providers (PHCPs). However, there is limited evidence on whether PHCPs’ level of knowledge and attitude can predict better HBV infection prevention practices. This study established the relationship between knowledge, attitude, and HBV infection prevention practices among PHCPs in Wakiso district, Central Uganda.

**Methods:** A cross-sectional study design was used. Data were collected from 306 PHCPs, using a structured questionnaire. PHCPs were randomly selected from 55 healthcare facilities. STATA version 14.0 was used to analyse data. A ‘modified Poisson’ regression model was used for inferential statistics.

**Results:** About 42.2% of PHCPs exhibited poor knowledge of HBV infection transmission and prevention, 41.8% had a negative attitude, and 41.5% exhibited poor prevention practices. Age (PR 1.82, 95% CI: 1.24–2.66) was positively associated with the level of knowledge. Healthcare facility level (PR 0.53, 95% CI: 0.34–0.84), main department of work (PR 0.69, 95% CI: 0.51–0.95), years in service (PR 0.66, 95% CI: 0.44–0.99), working in private not-for-profit healthcare facilities (PR 0.59, 95% CI: 0.34–0.99), and public healthcare facilities (PR 0.58, 95% CI: 0.42–0.80) were negatively associated with the level of knowledge. There was a negative association between the location of healthcare facility (PR 0.76, 95% CI: 0.62–0.93) and attitude, and a positive association between level of knowledge (PR 1.36, 95% 1.12–1.65) and attitude. Working in a public healthcare facility (PR 0.80, 95% CI: 0.64–0.99) was negatively associated with practices while having a positive attitude (PR 1.60, 95% CI: 1.28–1.99) predicted better HBV infection prevention practices.

**Conclusion:** PHCPs who were more knowledgeable about HBV infection were more likely to have a positive attitude. In turn, having a positive attitude was associated with better HBV infection prevention practices. There is a need to sensitise PHCPs on HBV infection, and provision of screening and vaccination services in order to address the KAP gaps.

## Background

Globally, hepatitis B virus (HBV) infection remains a serious public health problem, affecting over 257 million people (WHO, 2017a). The World Health Organization (WHO) estimates that over 887,000 people succumb to chronic HBV infection. These figures are comparable to deaths caused by tuberculosis (TB) and higher than those caused by Human Immune Virus (HIV). While TB and HIV-related deaths are on the decline, HBV infection-related deaths are on the rise (WHO, 2017a). The burden of chronic HBV infection is disproportionately higher in low and middle-income countries than in high-income countries (Stanaway et al., 2016). In Uganda, the seroprevalence of HBV infection among primary healthcare providers (PHCPs) at a tertiary hospital in 2010 was reported to be as high as 8.1%, while the prevalence of lifetime exposure among PHCPs was 48.1% (Ziraba, Bwogi, Namale, Wainaina, & Mayanja-Kizza, 2010).

PHCPs include all paid and unpaid individuals providing healthcare, working, or training in healthcare settings. Such individuals have a reasonably high risk of exposure to infectious materials, including blood or other body fluids, contaminated medical supplies and equipment, or contaminated environmental surfaces (Ray, 2017; Schillie et al., 2013). The nature of PHCPs’ work increases their risk of HBV infection, making it one of their major occupational risks (Auta et al., 2017; Kisic-Tepavcevic et al., 2017; Konlan, Aarah-Bapuah, Kombat, & & Wuffele, 2017). Exposure to HBV infection mainly results from needle stick and other sharp injuries, contact with blood and bodily fluids through scratches, abrasions, or burns on the skin as well as mucosal surfaces of the eyes, nose, or mouth through accidental splashes (WHO, 2017b). Once infected, PHCPs may either present asymptomatically or symptomatically, often resulting in acute or chronic infections such as hepatocellular necrosis, liver cirrhosis, inflammation and death (Lamberti et al., 2015; WHO, 2015).

The fact that PHCPs are a high-risk group, WHO guidelines on prevention of hepatitis B recommend infection control strategies, including good injection safety practices, vaccination, post-exposure prophylaxis, early diagnosis, and management of chronic HBV infection among healthcare providers (WHO, 2017b). However, these prevention strategies only remain to be desired in not only middle and high-income but also low-income settings (WHO, 2017b), largely due to inadequate knowledge, negative attitude and lack of prevention supplies such as testing kits and vaccines (Ishizaki et al., 2017; Konlan et al., 2017). Low levels of knowledge of HBV transmission and pre and post-exposure management have been reported among PHCPs, including Iranian medical specialists, nurses in Ghana, and medical and health science students in Ethiopia (Kabir et al., 2010; Konlan et al., 2017 Mesfin & Kibret, 2013;). However, there are no local context-specific data in Wakiso district and other parts of Uganda to inform policy and programing. Therefore, understanding the relationship between level of knowledge, attitude, and HBV infection prevention practices is critical for ensuring a healthy and vibrant health workforce (Maltezou & Poland, 2014; Ray, 2017). The present study therefore, assessed the relationship between level of knowledge, attitude and HBV infection prevention practices.

## Materials and methods

### Study area

This study was conducted in healthcare facilities in Wakiso district, located in the central region of Uganda. The district encircles Kampala, Uganda’s capital and business hub, and boarders Mpigi, Luweero, Nakaseke, and Mityana districts to the north; Mukono to the east and Kalangala district to the south. Wakiso is the most populated district in the country, with approximately 2,562,900 people (UBOS, 2018). Wakiso has 7 health sub-districts namely; Busiro East, Busiro North, Busiro South, Kyadondo East, Kyadondo North, Kyadondo South, and Entebbe municipality. The district has a total of 589 healthcare facilities which include 15 general hospitals (Ministry of Health, 2018a).

### Study design, sample size, sampling procedure, and data collection tool

A cross-sectional study design was used to obtain data from 306 PHCPs, randomly selected from 55 health facilities. Data collection was undertaken in July 2018. Hospitals, Health Centre IVs and IIIs, particularly those offering maternity services, were purposively selected. Healthcare facilities at these levels provide high-risk medical interventions such as blood transfusions, delivering mothers, and other surgical procedures that can elevate the risk of transmission of HBV infection.

Using a knowledge prevalence of 80% (Abdela, Woldu, Haile, Mathewos, & Deressa, 2016), a 95% level of confidence and a margin of error of 5%, we calculated a sample size of 246 PHCPs. Considering a design effect of 1.2, and a non-response rate of 10%, the total estimated sample size was 325. The sampling procedure used in the selection of study participants has comprehensively been described by Ssekamatte et al. (2020). Briefly, 6 general hospitals and 16 health centre IVs were purposively selected while 33 health centre IIIs were randomly selected from the Wakiso district healthcare facility inventory (Ministry of Health, 2018a). Data on PHCP KAP were collected using a researcher-developed questionnaire, which was informed by a review of relevant literature, and consultation with experts and researchers in hepatitis B infection prevention and management. The data collection tool included questions on socio-demographics, knowledge of HBV infection, attitude, and HBV infection prevention practices. Data obtained from PHCPs were entered using koboCollect, a mobile application. In order to avoid loss of the survey data on mobile phones, data were uploaded to the server on a daily basis by the research assistants. For quality assurance and control purposes, all research assistants underwent a four-day training prior to data collection in order to familiarise with the data collection tool. A total of 16 healthcare providers, selected from two primary healthcare facilities in Kampala city were interviewed during the validation of the study tool. Besides, the mobile data collection tool was designed with the necessary skips and restrictions in order to minimise errors, incorrect entries, and omissions. Data were checked for quality and completeness prior to submission to the online server.

### Study variables

The independent variables included PHCPs’ socio-demographics, such as age, sex, level of education, and work-related variables such as level of healthcare facility where the PHCP was working, ownership of the healthcare facility where the PHCP was working, main department of work, history of needle stick injury, and the years in service. The main departments of work included inpatient, maternity and outpatient inpatient departments. The inpatient department comprised of the theatre and the different wards (male, female, and children’s wards), the outpatient department comprised of the laboratory, dental, radiology, nutrition, and counselling clinics while the maternity department comprised of the labour suite, antenatal and postnatal wards and the delivery rooms. The dependent variables included level of knowledge, attitude and HBV infection prevention practices.

### Measurement of the level of knowledge of HBV infection

A total of 16 questions were used to determine the knowledge of hepatitis B. Each question was scored as indicated in Table 1. The total knowledge score for each study participant was obtained by summing up scores obtained from each question. The maximum knowledge score a PHCP would attain was 43.0.

**Table 1.** Questions used for measurement of knowledge.

No.	Knowledge questions	Possible responses and score
1	Have you ever heard about HBV infection?	Yes = 1, No = 0
2	Do you know the causative agent for HBV infection?	Yes = 1, No = 0
3	How can HBV infection be transmitted?	Each correct response was scored 1 point up to a maximum of 5 points while a wrong response was scored 0
4	Can HBV infection be transmitted by carriers?	Yes = 1, No = 0
5	Which populations are at an increased risk of contracting HBV infection?	Each correct response was scored 1 point up to a maximum of 5 points while a wrong response was scored 0
6	What practices increase the risk of HBV infection?	Each correct response was scored 1 point up to a maximum of 6 points while a wrong response was scored 0
7	What are the clinical signs and symptoms of HBV infection?	Each correct response was scored 1 point up to a maximum of 7 points while a wrong response was scored 0
8	What complications are related to HBV infection?	Each correct response was scored 1 point up to a maximum of 5 points while a wrong response was scored 0
9	Is HBV infection treatable?	Yes = 1, No = 0
10	Is HBV infection preventable?	Yes = 1, No = 0
11	Can antiretroviral drugs and supportive treatments be used for the management of HBV infection?	Yes = 1, No = 0
12	How can HBV infection be prevented?	Each correct response was scored 1 point up to a maximum of 5 points while a wrong response was scored 0
13	Are you aware of the HBV infection immune response test?	Yes = 1, No = 0
14	What is the recommended hepatitis B vaccine dose?	3 vaccine doses were scored 1 point while a wrong response was scored 0
15	Does HBV infection have post-exposure prophylaxis?	Yes = 1, No = 0
16	What is used as post-exposure prophylaxis for HBV infection?	Hepatitis B vaccine/ immunoglobulin was scored 1 point while a wrong response was scored 0

### Measurement of attitude towards hepatitis B prevention strategies

A total of 8 questions were used to determine attitude towards hepatitis B prevention strategies. PHCPs were asked if, they were at an elevated risk of contracting hepatitis B (Yes = 1, No = 0); it is important to know the hepatitis B status of patients (Yes = 1, No = 0); healthcare providers can infect patients with the hepatitis B virus (Yes = 1, No = 0); it is necessary to get vaccinated against hepatitis B (Yes = 1, No = 0); Hepatitis B vaccine is safe (Yes = 1, No = 0); Hepatitis B vaccine is effective (Yes = 1, No = 0); level of effectiveness of the Hepatitis B vaccine (not effective at all = 0, slightly effective = 1, very effective = 2); following infection control guidelines is important in reducing hepatitis B infections in healthcare settings (Yes = 1, No = 0). The total attitude score for each study participant was obtained by summing up scores obtained from each question. The maximum attitude score a PHCP would attain was 9.0.

### Measurement of hepatitis B prevention practices

A total of 11 questions were used to determine PHCPs’ Hepatitis B prevention practices. PHCPs were asked; (1) how many hepatitis B vaccines they had ever received (No dose = 0, 1 dose = 1, 2 doses = 2, 3 doses = 3); (2) whether they had completed the 3 hepatitis B vaccine doses (Yes = 1, No = 0); (3) if they had ever taken a hepatitis B surface antigen test (HBsAg) (Yes = 1, No = 0); (4) whether they had ever done a Hepatitis B immune response test (Yes = 1, No = 0); (5) if they had ever done a hepatitis B core antibody test (HBcAb) (Yes = 1, No = 0); (6) if they had ever taken a hepatitis B surface antibody test (Anti-HBs or HBsAb) (Yes = 1, No = 0); (7) if they always follow infection control guidelines (Yes = 1, No = 0); (8) whether they had a needle stick injury in the last 12 months (No = 1, Yes = 0); (9) if they had ever attended any health-related program on Hepatitis B (Yes = 1, No = 0); (10) whether they always use a new syringe for each procedure (do not reuse syringes) (Yes = 1, No = 0) and (11) if they had ever received any training on post-exposure prophylaxis for hepatitis B (Yes = 1, No = 0). The total practice score for each study participant was obtained by summing up scores obtained from each question. The maximum practice score a PHCP could attain was 13.0.

### Data management and statistical analyses

Data were collected using the KoboCollect mobile application and exported to STATA 14.0 for statistical analysis. The data set was password-protected and only accessible to the data management team. Prior to data analysis, data cleaning was performed to minimise errors. A Linear regression model was the preferred model for the analysis of the variation in the knowledge, attitude and practice scores among PHCPs, however, normality tests conducted using the Shapiro–Wilk test and constant variance tests using Breusch–Pagan test indicated that data violated the underlying assumptions of the model (Dunn & Smyth, 2018 Hazra & Gogtay, 2016; Schmidt & Finan, 2018;). Therefore, knowledge, attitude, and practice scores were categorised using the median at the cutoff (Mursy & Mohamed, 2019).

The level of knowledge was categorised as low if a PHCP scored below the median mark and high if a respondent scored the median mark and above. Attitude towards hepatitis B prevention strategies was categorised as negative if a PHCP scored below the median mark and positive if a respondent scored the median or above it. Practices were classified as poor if a PHCP scored below the median mark or good if a PHCP scored the median and above. The prevalence of the outcomes was above 10%, and therefore, prevalence ratios generated by a generalised linear ‘modified Poisson’ regression model were used to measure the strength of association between the independent and the dependent variables (Martinez et al., 2017). For all dependent variables, bivariable analysis was first conducted using a ‘modified Poisson’ regression model, and variables with a *p*-value less than 0.2 at bivariable analysis were then included in the multivariable model (Gulilat & Tiruneh, 2014; Yelland, Salter, & Ryan, 2011). Although age and sex were not significant in the bivariable analysis, these variables were included in the multivariable model because they are known to be important clinical confounders (Pourhoseingholi, Baghestani, & Vahedi, 2012; Yelland et al., 2011).

### Ethics statement

Ethical approval was obtained from the Makerere University School of Public Health Higher Degrees and Research Ethics Committee (MakSPH HDREC). Permission to interview PHCPs was obtained from the Wakiso District Health Office and the respective administration of participating healthcare facilities. Informed written consent was obtained from all study participants. In addition, the principles of respect, beneficence, and justice were observed in the selection of study participants. Participation in this study was voluntary.

## Results

### Socio-demographic characteristics of respondents

Data were collected and analysed for 306 PHCPs (response rate was 94.1%), of whom 67.3% (206/306) were female; 67.6% (207/306) were aged between 20–30 years, and 58.2% (178/306) were single. About 15.4% (47/306) of the PHCPs were enrolled nurses, and 15.4% (84/306) of the PHCPs were working with the maternity department. In addition, 64.7% (198/306) of PHCPs had practiced for less than 5 years (Table 2).

**Table 2.** Socio-demographic characteristics of the respondents.

Variable	Attribute	Frequency (*n* = 306)	Percentage (%)
Sex of the respondents	Female	206	67.3
	Male	100	32.7
Age category (Mean age = 29.5 ± 7.7)	20–30	207	67.6
	31–40	70	22.9
	41 and above	29	9.5
Marital status	Married	128	41.8
	Single	178	58.2
Level of healthcare facility	Healthcentre III	133	43.5
	Healthcentre IV	120	39.2
	Hospital	53	17.3
Ownership of healthcare facility	Private for Profit (PFP)	136	44.4
	Private Not-For-Profit (PNFP)	30	9.8
	Public	140	45.8
Cadre	Clinical officer	37	12.1
	Enrolled midwife	45	14.7
	Enrolled nurse	47	15.4
	Laboratory assistant	31	10.1
	Laboratory technician/technologist	43	14.1
	Medical doctor	10	3.3
	Nursing assistant	20	6.5
	Dental officer	15	4.9
	Registered midwife	21	6.9
	Registered nurse	30	9.8
	Theatre assistant	2	0.7
	Other	5	1.6
Main department of work	Maternity	84	15.4
	In patient department	47	27.5
	Outpatient department	175	57.1
Years in service	Below 5 years	198	64.7
	Above 5 years	108	35.3

### Knowledge on HBV infection

A vast majority, 95.1% (291/306) of the PHCPs knew the recommended hepatitis B vaccine dose. Only 58.2 (178/306) of the PHCPs knew that HBV infection can be transmitted through sexual contact; 21.9% (67/306) knew that unsafe injection practices increase the risk of HBV infection and 25.8 (79/306) knew that liver cirrhosis can be associated with HBV infection. Only 3.9% (12/306) knew that HBV infection could be prevented by safe injection practices including minimum use of syringes, and only 48% (147/306) knew that HBV infection has post-exposure prophylaxis (Table 3).

**Table 3.** General knowledge of Hepatitis B among primary healthcare providers in Wakiso district, Central Uganda.

Knowledge variables	Satisfactory response (*n*) (%)
**Awareness about hepatitis B**	
Ever heard about hepatitis B	306 (100)
Knowledgeable about the causative agent of hepatitis B	293 (95.8)
**How can hepatitis B be transmitted?**^a^	
Sexual contact	178 (58.2)
Mother to child	140 (45.8)
Blood transfusion	165 (53.9)
Through sharing sharps	163 (53.3)
Through contact with body fluids	44 (14.4)
Can hepatitis B be transmitted by carriers?	257 (84)
**Which populations are at an increased risk of contracting hepatitis B**^a^	
Female sex workers	209 (68.3)
Men who have sex with men	36 (11.8)
Injecting drug and substance users	38 (12.4)
Long truck drivers	24 (7.8)
Waste handlers	51 (16.7)
**What practices increase the risk of hepatitis B infection?**^a^	
Sharing sharps	71 (23.2)
Unsafe injection practices	67 (21.9)
Unsafe waste handling	70 (22.9)
Unsafe blood transfusion	90 (29.4)
Limited use of protective gears	147 (48.0)
Low vaccination status	14 (4.6)
**What are the clinical signs and symptoms of hepatitis B?**^a^	
Fever	198 (64.7)
Vomiting	57 (18.6)
Jaundice	256 (83.7)
Abdominal discomfort	152 (49.7)
Clay coloured stool	9 (2.9)
Convulsions	4 (1.3)
Dark coloured urine	74 (24.2)
**What complications are related to hepatitis B viral infection?**^a^	
Acute hepatitis B disease	5 (1.6)
Liver cirrhosis	79 (25.8)
Liver cancer	34 (11.1)
Anaemia	17 (5.6)
Hepatic encephalopathy	16 (5.2)
**Treatment of hepatitis B**	
Is hepatitis B treatable?	270 (88.2)
Is hepatitis B preventable?	305 (99.7)
Can antiretroviral drugs and supportive treatments be used for the management of hepatitis B?	220 (71.8)
**How can hepatitis B be prevented?**^a^	
Screening of blood before transfusion	57 (18.6)
Vaccination against HBV	273 (89.2)
Use of protective gears like gloves	215 (70.3)
Safe waste management	54 (17.6)
Safe injection practices (including minimum use of syringes)	12 (3.9)
**Ever heard about HBV immune response test**	75 (24.5)
**Know the recommended dose for hepatitis B vaccine**	291 (95.1)
**Know that hepatitis B has post-exposure propylaxis**	147 (48.0)
**Know that hepatitis B immunoglobulin is used for post-exposure propylaxis**	19 (6.2)

^a^ Multiple response.

### Attitude and HBV infection prevention practices

Among the 306 PHCPs, 74.8% (229/306) mentioned that the hepatitis B vaccine was very effective; 98.4% (301/306) felt that they were at an elevated risk of contracting HBV infection; 95.4% (292/306) thought that it was important to know the HBV status of the patient prior to being treated while 96.4% (295/306) thought that healthcare providers could infect patients with HBV infection. Almost all PHCPs, 99.7% (305/306) felt it was necessary to get vaccinated against HBV infection. Approximately 7.8% (24/306) felt that the hepatitis B vaccine was not safe, while 6.2% (19/306) thought the vaccine was not effective. Furthermore, 90.2% (276/306) felt that following infection control guidelines could safeguard them against HBV infection (Table 4).

**Table 4.** Attitude and practices towards Hepatitis B prevention among primary healthcare providers in Wakiso district, Central Uganda.

Variable	Satisfactory response (*N*) (%)
**Attitude towards hepatitis B prevention strategies**	
PHCPs are at an elevated risk of contracting hepatitis B infection	301 (98.4)
It is important to know hepatitis B status of the patient before treatment	292 (95.4)
PHCPs can infect patients with hepatitis B	295 (96.4)
It is necessary to get vaccinated against hepatitis B	305 (99.7)
Hepatitis B vaccine is safe	282 (92.2)
Hepatitis B vaccine is effective	287 (93.8)
**To what extent is the hepatitis B vaccine effective?**	
Not effective	27 (8.8)
Slightly effective	50 (16.4)
Very effective	229 (74.8)
Following infection control guidelines can safeguard PHCPs against HBV infection	276 (90.2)
**Hepatitis B prevention Practices**	
Number of hepatitis B vaccine doses received	
No dose	64 (20.9)
1 dose	24 (7.8)
2 doses	41 (13.4)
3 doses	177 (57.8)
Completed 3 doses of hepatitis B	177 (57.8)
Ever taken a hepatitis B surface antigen test (HBsAg)	260 (84.9)
Ever done a Hepatitis B immune response test	3 (1.0)
Ever done hepatitis B core antibody test (HBcAb)	0 (0.0)
Ever taken a hepatitis B surface antibody test (Anti-HBs or HBsAb)	3 (1.0)
Follow infection control guidelines at the health facility	267 (87.3)
Did not have a needle stick injury in the last 12 months	257 (84.0)
Ever attended a health-related program on Hepatitis B	60 (19.6)
Use a new syringe for each procedure (do not reuse syringes)	302 (98.6)
Ever trained on post-exposure prophylaxis for hepatitis B	16 (5.2)

Regarding HBV infection prevention practices, only 57.8% (177/306) had completed the recommended three vaccine doses. Almost all, 99% (303/306) of the PHCPs had never done a hepatitis B immune response test, and 87.3% (267/306) reported following infection control guidelines at their health facility. About 16% (49/306), PHCPs reported having been exposed to a needle stick injury in the last 12 months. Only 19.6% (60/306) had ever attended a health-related training on hepatitis B prevention. Only 5.2% (16/306) had ever received training on post-exposure prophylaxis (Table 4).

### Level of knowledge, attitude, and HBV infection prevention practices

The Linear regression models fitted for the knowledge, attitude and practices scores violated the assumptions of normality and constant variance with Shapiro–Wilk and Breusch–Pagan test results of (*p* < 0.001, *p* = 0.026), (*p* < 0.001, *p* < 0.001) and (*p* < 0.001, *p* = 0.012), respectively. The median knowledge score was 16.0 (IQR = 14, 18). The maximum knowledge score attained by the study participants was 30.0, while the minimum was 8.0. About 42.2% (129/306) of the PHCPs had a low level of knowledge of hepatitis B prevention.

The median attitude score was 9.0 (IQR = 8, 9). The maximum attitude score attained by the study participants was 9.0, while the minimum was 4.0. Over 41.8% (128/306) had negative attitudes towards hepatitis B prevention strategies. The median practice score was 7.0 (IQR = 5, 8). The minimum practice score attained by the study participants was 1.0 while the maximum was 10.0. Over 41.5% (179/306) of the PHCPs exhibited poor hepatitis B prevention strategies. These median score values informed the subsequent dichotomisation of the knowledge, attitude and practices variables.

### Factors associated with the level of knowledge on hepatitis B among primary healthcare providers in Wakiso district, Central Uganda

After adjusting for sex, age group and level of healthcare facility where the PHCP was working, only the main department of work and ownership status of the healthcare facility was significantly associated with the level of knowledge. PHCPs working in public healthcare facilities had a 32% lower prevalence of knowledge about HBV infection transmission and prevention compared to their counterparts in private for profit healthcare facilities (PR 0.68, 95% CI: 0.55–0.84). PHCPs working in the outpatient department or clinic had a 23% lower prevalence of knowledge about HBV infection transmission and prevention compared to their counterparts in the inpatient department (PR 0.77, 95% CI: 0.61–0.97). Similarly, PHCPs working in the maternity department were less knowledgeable about HBV infection transmission and prevention compared to their counterparts in the inpatient department (PR 0.75, 95% CI: 0.56–0.99) (Table 5).

**Table 5.** Level of knowledge of hepatitis B and associated factors among primary healthcare providers in Wakiso district, Uganda.

Variable	Attribute	Freq *n*	Level of knowledge		Unadjusted PR (95% CI)	Adjusted PR (95% CI)	*p*-value
			High *n* (%)	Low *n* (%)			
**Sex**	Female	206	112 (63.3)	94 (72.9)	1.0	1.0	
	Male	100	65 (36.7)	35 (27.1)	1.19 (0.98–1.44)	1.20 (0.97–1.47)	0.082
**Age group**	20–30	207	123 (69.5)	84 (65.1)	1.0	1.0	
	31–40	70	39 (22.0)	31 (24.0)	0.93 (0.73–1.18)	0.99 (0.80–1.24)	0.988
	41 and above	29	15 (8.5)	14 (10.9)	0.87 (0.60–1.25)	1.98 (0.67–1.43)	0.081
**Level of healthcare facility**	Health Centre III	133	69 (39.0)	64 (49.6)	1.0	1.0	
	Health Centre IV	120	80 (45.2)	40 (31.0)	1.28 (1.04–1.58)	**1.03** (0.83–1.29)	0.739
	Hospital	53	28 (15.8)	25 (19.4)	1.01 (0.75–1.37)	0.83 (0.62–1.12)	0.229
**Ownership of Health Facility**	Private for profit	136	98 (55.4)	38 (29.5)	1.0	1.0	
	Private not for profit	30	11 (6.2)	19 (14.7)	0.50 (0.31–0.82)	0.51 (0.32–0.81)	**0**.**005**
	Public	140	68 (38.4)	72 (55.8)	0.67 (0.55–0.82)	0.68 (0.55–0.84)	***P* < 0.001**
**Location of Health facility**	Rural	102	57 (32.2)	45 (34.9)	1.0		
	Urban	204	120 (67.8)	82 (65.1)	1.05 (0.85–1.29)		
**Marital status**	Married	128	69 (39.0)	59 (45.7)	1.0		
	Single	178	108 (61.0)	70 (54.3)	1.12 (0.92-1.37)		
**Main department of work**	Inpatient clinic (excludes maternity)	47	35 (19.8)	12 (9.3)	1.0	1.0	
	Maternity ward	84	42 (23.7)	42 (32.6)	0.67 (0.51–0.88)	0.75 (0.56–0.99)	**0**.**048**
	Outpatient clinic	175	100 (56.5)	75 (58.1)	0.76 (0.62–0.94)	0.77 (0.61–0.97)	**0**.**030**
**Years in service**	Above 5	108	59 (33.3)	49 (38.0)	1		
	Below 5	198	118 (66.7)	80 (62.0)	1.09 (0.88–1.34)		

### Factors associated with PHCPs’ attitude toward hepatitis B prevention strategies

After adjusting for sex, age and ownership of the healthcare facility where the PHCP was working, only the location of the healthcare facility, and the level of knowledge of PHCPs were statistically associated with attitude towards HBV infection prevention strategies. Prevalence of a positive attitude towards HBV infection transmission and prevention was lower among PHCPs working in a healthcare facility located in an urban setting compared to their counterparts working in a healthcare facility located in a rural setting (PR 0.78, 95% CI: 0.64–0.95, *p* = 0.017). PHCPs with a high level of knowledge had a 36% higher prevalence of a positive attitude towards HBV infection prevention strategies compared to those with a low level of knowledge (PR 1.36, 95% 1.10–1.69, *p* = 0.004) (Table 6).

**Table 6.** Attitude towards HBV infection prevention and associated factors among primary healthcare providers in Wakiso district, Uganda.

Variable	Attribute	Freq *n*	Attitude		Unadjusted PR (95% CI)	Adjusted PR (95% CI)	*p*-value
			Positive *n* (%)	Negative *n* (%)			
**Sex**	Female	206	120 (67.4)	86 (67.2)	1.0	1.0	
	Male	100	58 (32.6)	42 (32.8)	0.99 (0.81–1.22)	0.94 (0.77–1.15)	0.602
**Age group**	20–30	207	119 (66.9)	88 (68.8)	1.0	1.0	
	31–40	70	43 (24.2)	27 (21.1)	1.06 (0.85–1.33)	1.09 (0.88–1.36)	0.406
	41 and above	29	16 (9.0)	13 (10.2)	0.95 (0.67–1.36)	1.03 (0.73–1.46)	0.830
**Level of Health Facility**	Health Centre III	133	83 (46.6)	50 (39.1)	1.0		
	Health Centre IV	120	66 (37.1)	54 (42.2)	0.88 (0.71–1.08)		
	Hospital	53	29 (16.3)	24 (18.7)	0.87 (0.66–1.15)		
**Ownership of Health Facility**	Private for profit	136	85 (47.8)	51 (39.8)	1.0	1.0	
	Private not for profit	30	18 (10.1)	12 (9.4)	0.96 (0.69–1.32)	1.15 (0.83–1.59)	0.390
	Public	140	75 (42.1)	65 (50.8)	0.85 (0.70–1.04)	0.86 (0.69–1.07)	0.183
**Location of Health facility**	Rural	102	67 (37.6)	35 (27.3)	1.0	1.0	
	Urban	204	111 (62.4)	93 (72.7)	0.82 (0.68–1.00)	0.78 (0.64–0.95)	**0**.**017**
**Marital status**	Married	128	77 (43.3)	51 (39.8)	1.0		
	Single	178	101 (56.7)	77 (60.2)	0.94 (0.77–1.14)		
**Main department of work**	Inpatient clinic	47	29 (16.3)	18 (14.1)	1.0		
	Maternity ward	84	52 (29.2)	32 (25.0)	1.00 (0.75–1.32)		
	Outpatient clinic	175	97 (54.5)	78 (60.9)	0.89 (0.69–1.16)		
**Years in service**	Below 5	198	114 (64.0)	84 (65.6)	1.0		
	Above 5	108	64 (36.0)	44 (34.4)	1.02 (0.84–1.25)		
**Knowledge level**	Low	129	62 (34.8)	67 (52.3)	1.0	1.0	
	High	177	116 (65.2)	61 (47.7)	1.36 (1.10–1.68)	1.36 (1.10–1.69)	**0**.**004**

### Factors associated with PHCPs’ Hepatitis B prevention practices

After adjusting for sex, age, marital status, years in service, ownership and location of the healthcare facility where the PHCP was working, and the level of knowledge of HBV infection, the only factor significantly associated with good practices was a positive attitude towards hepatitis B prevention strategies. PHCPs with a positive attitude had a 59% higher prevalence of exhibiting good HBV infection prevention practices (PR 1.59, 95% CI: 1.28–1.98, *p* < 0.001) compared to those with a negative attitude (Table 7).

**Table 7.** Hepatitis B-related practices and associated factors among primary healthcare providers in Wakiso district, Uganda.

Variable	Attribute	Freq *n*	Practices		Unadjusted PR (95% CI)	Adjusted PR (95% CI)	*p*-value
			Good *n* (%)	Poor *n* (%)			
**Sex**	Female	206	117 (65.4)	89 (70.1)	1.0	1.0	
	Male	100	62 (34.6)	38 (29.9)	1.09 (0.89–1.32)	1.00 (0.82–1.21)	0.987
**Age group**	20–30	207	127 (70.9)	80 (63.0)	1.0	1.0	
	31–40	70	37 (20.7)	33 (26.0)	0.86 (0.67–1.10)	1.02 (0.75–1.40)	0.855
	41 and above	29	15 (8.4)	14 (11.0)	0.84 (0.58–1.21)	1.06 (0.69–1.64)	0.774
**Level of Health Facility**	Health Centre III	133	75 (41.9)	58 (45.7)	1.0		
	Health Centre IV	120	69 (38.5)	51 (40.2)	1.01 (0.82–1.26)		
	Hospital	53	35 (19.6)	18 (14.2)	1.17 (0.91–1.49)		
**Ownership of Health Facility**	Private for profit	136	90 (50.3)	46 (36.2)	1.0	1.0	
	Private not for profit	30	22 (12.3)	8 (6.3)	1.10 (0.86–1.41)	1.10 (0.85–1.42)	0.439
	Public	140	67 (37.4)	73 (57.5)	0.72 (0.58–0.89)	0.80 (0.65–1.00)	0.054
**Location of Health facility**	Rural	102	52 (29.1)	50 (39.4)	1.0	1.0	
	Urban	204	127 (70.9)	77 (60.6)	1.22 (0.58–1.51)	1.18 (0.95–1.47)	0.117
**Marital status**	Married	128	69 (38.5)	59 (46.5)	1.0	1.0	
	Single	178	110 (61.5)	68 (53.5)	1.14 (0.94–1.39)	1.07 (0.86–1.33)	0.531
**Main department of work**	Inpatient clinic	47	30 (16.8)	17 (13.4)	1.0		
	Maternity ward	84	49 (27.4)	35 (27.5)	0.91 (0.68–1.21)		
	Outpatient clinic	175	100 (55.8)	75 (59.1)	0.89 (0.69–1.15)		
**Years in service**	Below 5	198	123 (68.7)	75 (59.1)	1.0	1.0	
	Above 5	108	56 (31.3)	52 (40.9)	0.83 (0.67–1.03)	0.87 (0.65–1.17)	0.377
**Knowledge level**	Low	129	66 (36.9)	63 (49.6)	1.0	1.0	
	High	177	113 (63.1)	64 (50.4)	1.24 (1.01–1.52)	1.11 (0.90–1.30)	0.135
**Attitude**	Negative	128	55 (30.7)	73 (57.5)	1.0	1.0	
	Positive	178	124 (69.3)	54 (42.5)	1.62 (1.29–2.02)	1.59 (1.28–1.98)	***P *< 0.001**

## Discussion

The current study established the level of knowledge, attitude, and HBV infection prevention practices among PHCPs. Slightly more than half of the PHCPs had a high level of knowledge of transmission and prevention of HBV infection, while 4 in 10 had a negative attitude and exhibited poor hepatitis B prevention practices. The PHCP’s level of knowledge was associated with a positive attitude, and in turn, a positive attitude was associated with better Hepatitis B prevention strategies. However, a high level of knowledge did not translate into better Hepatitis B prevention strategies.

This study revealed that a significant proportion of PHCPs had a low level of knowledge of transmission and prevention of HBV infection. The low level of knowledge of HBV infection among PHCPs in this study is a big challenge given that all PHCPs are expected to have adequate knowledge of prevention and transmission of HBV infection (Abeje & Azage, 2015). A high level of knowledge of transmission and prevention of the HBV infection among PHCPs is vital for the prevention of infections in the community since PHCPs are expected to sensitise the community, which is otherwise expected to have a lower level of knowledge. A high level of knowledge is also critical in the prevention of HBV infections in a healthcare setting. The proportion of PHCPs in this study who had a higher level was slightly higher than that reported among healthcare providers in Djoungolo Health District, Cameroon. The difference in the findings could have been due to the smaller sample size and lower response rate (61.34%) reported by Tatsilong et al. (2016). The low level of knowledge reported in our study may also be attributed to the few trainings on prevention and transmission of HBV infection conducted among PHCPs in the study area. After adjusting for confounding, only the main department of work and the location of the healthcare facility where the PHCP was working were significantly associated with the level of knowledge. PHCPs working in public and private not-for-profit healthcare facilities had a lower level of knowledge of hepatitis B prevention compared to their counterparts in private for profit healthcare facilities. This could be so because it is mainly the private for profit healthcare facilities that provide HBV infection screening and vaccination services to adults. Therefore, this could trigger PHCPs in private for profit healthcare facilities to seek more information about the disease because they are likely to face more questions from clients who seek HBV infection screening and vaccination services. These findings therefore, highlight the need for health authorities to sensitise PHCPs in public and private, as well as those in rural healthcare facilities on hepatitis B transmission and prevention strategies.

Our study also revealed that working in the outpatient department or in the maternity ward was a significant predictor of a low level of knowledge of transmission and prevention of hepatitis B. This may have resulted from PHCPs in these departments not being involved in trainings on HBV infection. PHCPs working in the inpatient department may have been prioritised to attend such trainings, thus a higher likelihood of being knowledgeable about HBV infection. None the less, the risk of HBV infection is relatively high in maternity and casualty wards due to spilled blood and body fluids during delivery (Adjei, Asamoah, Atibila, Ti-Enkawol, & Ansah-Nyarko, 2016). Our findings thus reveal the need to train PHCPs in the outpatient and maternity department given that they are also at an elevated risk.

Nearly 42% of PHCPs had a negative attitude towards hepatitis B prevention strategies. These findings are, however, not in agreement with a study conducted by Akibu, Nurgi, Tadese, and Tsega (2018), which indicated that over 77.8% of PHCPs in a healthcare setting in Ethiopia had a positive attitude towards the prevention of hepatitis B. The low proportion of PHCPs with a positive attitude towards hepatitis B prevention strategies in our study could be a result of the few hepatitis B focused trainings. Most occupational exposure trainings among PHCPs focus on infection prevention and control in general, thereby limiting attention accorded to HBV infection. However, trainings focused on hepatitis B transmission and prevention have the potential to improve PHCPs’ attitude towards hepatitis B prevention strategies (Dunn & Smyth, 2018). PHCPs in our study setting may also have lost confidence in the safety and efficacy of the hepatitis B vaccine given the widespread sale of falsified vaccines in the country (National Drug Authority, 2018).

PHCPs working in urban healthcare facilities were less likely to have a positive attitude towards hepatitis B prevention strategies compared to their counterparts in rural healthcare facilities. This could be attributed to the underestimated hepatitis B susceptibility and severity among urban dwellers including PHCPs, which has been reported in previous studies (Stanaway et al., 2016; WHO, 2017a). Besides, PHCPs in urban settings, unlike those in rural healthcare facilities, have a heavy workload (Mæstad, Torsvik, & Aakvik, 2010), which makes it difficult for them to uptake hepatitis B prevention strategies (Chitimwango, 2017). Failure to uptake these strategies exposes them to HBV. However, exposure to HBV at times does not advance into the chromic form, which is associated with more serious health effects (Shi & Shi, 2009). This could make them reluctant to uptake preventive strategies. In contrast, PHCPs in rural healthcare facilities have a lower probability of exposure to infections due to a lower workload. Consequently, PHCPs in rural settings often strive to adhere to preventive strategies. This study demonstrates the need to change the attitude of the PHCPs in urban healthcare facilities towards the available Hepatitis B prevention strategies. This can be achieved through conducting regular continuous medical education on hepatitis B.

This study revealed that more than half (58.5%) of the PHCPs exhibited good hepatitis B prevention practices. The low proportion of PHCPs exhibiting good hepatitis B prevention practices could be as a result of the low investment in hepatitis B infection as an occupational risk for PHCPs, particularly in Central Uganda. Although Uganda has been commended for her efforts in the fight against Hepatitis B (Ministry of Health, 2018b), there has been little attention to improving PHCPs’ access to a wide range of prevention services such as screening and vaccination. Our study further revealed that better hepatitis B prevention practices were associated with a positive attitude, and a positive attitude in this study was predicted by a higher level of knowledge. On the contrary, we did not find a significant association between knowledge and practices. Knowledge is known to influence the uptake of prevention strategies, such as vaccination and screening (Adekanle, Ndububa, Olowookere, Ijarotimi, & Ijadunola, 2015; Said & Jou, 2014; Ssekamatte et al., 2020). However, limited access to such services implies that PHCPs cannot uptake them. Therefore, the correlation of knowledge is only limited to a better attitude. Our findings imply that PHCPs who may want to uptake preventive services such as screening and vaccination are not able to get them.

The positive association between attitude and practice observed in this study could be related to the belief that the hepatitis B vaccine is safe and efficacious. Hepatitis B vaccine has widely been documented to be efficacious in the prevention of chronic hepatitis B infection (Ssekamatte et al., 2020; Van Damme et al., 2010). Such a belief might have motivated PHCPs to take an extra step in adhering to preventive strategies and uptaking screening and vaccination services. Our findings underscore the importance of capacity building as a strategy for improving PHCPs’ attitude. Once the attitude is improved, PHCPs are likely to uptake prevention services if availed (Akibu et al., 2018a)

### Study limitations

The study relied on self-reports with the possibility that the findings would have been affected by a recall bias. To avert this challenge, a one-year period was used with the assumption that PHCPs were in position to recall an occurrence within this period. Regarding hepatitis B-related practices, the study relied on self-reports to document whether the healthcare provider had received any vaccine dose. The study had a limited scope; therefore, antibody tests were not conducted. The findings of this study may not be generalised to all PHCPs in Uganda since the study was conducted in one district. Furthermore, as a cross-sectional study design, we could not establish a temporal causation effect.

## Conclusion and recommendations

Having a high level of knowledge was associated with a positive attitude. In turn, a positive attitude was associated with better hepatitis B prevention practices. This study underscores the need to sensitise PHCPs, especially those in rural healthcare facilities, as well as those in the outpatient and maternity departments since they had the lowest level of knowledge HBV infection prevention strategies. The government of Uganda also needs to scale up the provision of screening and vaccination services, most especially for PHCPs in PNFP and public healthcare facilities in Wakiso district. There is also a need to conduct a national-wide study to further understand the PHCP level of knowledge, attitude, and practices towards hepatitis B prevention. This would form a basis for informing policy. In addition, there is a need to establish the readiness of health facilities to provide hepatitis B prevention services such as screening and vaccination, and PHCP awareness about post-exposure prophylaxis for Hepatitis B.

## Data Availability

The datasets used during this study are available from the corresponding author upon reasonable request.

## Abbreviations

**Abbreviations:** HBV, Hepatitis B Virus; KAP, Knowledge, attitude and practice; PHCPs, Primary healthcare providers.

## References

[CIT0001] Abdela, A., Woldu, B., Haile, K., Mathewos, B., & Deressa, T. (2016). Assessment of knowledge, attitudes and practices toward prevention of hepatitis B virus infection among students of medicine and health sciences in Northwest Ethiopia. *BMC Research Notes*, *9*, 410. doi: 10.1186/s13104-016-2216-y 27543117 PMC4992214

[CIT0002] Abeje, G., & Azage, M. (2015). Hepatitis B vaccine knowledge and vaccination status among health care workers of Bahir Dar city administration, Northwest Ethiopia: A cross sectional study. *BMC Infectious Diseases*, *15*, 30. doi: 10.1186/s12879-015-0756-8 25637342 PMC4324798

[CIT0003] Adekanle, O., Ndububa, D. A., Olowookere, S. A., Ijarotimi, O., & Ijadunola, K. T. (2015). Knowledge of hepatitis B virus infection, Immunization with hepatitis B vaccine, risk perception, and challenges to control hepatitis among hospital workers in a Nigerian tertiary hospital. *Hepatitis Research and Treatment*, *2015*, 439867. doi: 10.1155/2015/439867 25685549 PMC4320901

[CIT0004] Adjei, C. A., Asamoah, R., Atibila, F., Ti-Enkawol, G. N., & Ansah-Nyarko, M. (2016). Mother-to-child transmission of hepatitis B: Extent of knowledge of physicians and midwives in eastern region of Ghana. *BMC Public Health*, *16*, 537. doi: 10.1186/s12889-016-3215-6 27401399 PMC4939625

[CIT0005] Akibu, M., Nurgi, S., Tadese, M., & Tsega, W. D. (2018a). Attitude and vaccination status of healthcare workers against hepatitis B infection in a teaching hospital, Ethiopia. *Scientifica*, *2018*, 6705305. doi: 10.1155/2018/6705305 29808158 PMC5901831

[CIT0006] Auta, A., Adewuyi, E. O., Tor-Anyiin, A., Aziz, D., Ogbole, E., Ogbonna, B. O., & Adeloye, D. (2017). Health-care workers’ occupational exposures to body fluids in 21 countries in Africa: Systematic review and meta-analysis. *Bulletin of the World Health Organization*, *95*, 831–841F. doi: 10.2471/BLT.17.195735 29200524 PMC5710084

[CIT0007] Chitimwango, P. C. (2017). *Knowledge, attitudes and practices of nurses in infection prevention and control within a tertiary hospital in Zambia*. Stellenbosch: Stellenbosch University.

[CIT0008] Dunn, P. K., & Smyth, G. K. (2018). *Linear regression models: Diagnostics and model-building. Generalized linear models with examples in R*. New York; Springer. 2018 Nov 10.

[CIT0009] Gulilat, K., & Tiruneh, G. (2014). Assessment of knowledge, attitude and practice of health care workers on infection prevention in health institution Bahir Dar city administration. *Science Journal of Public Health*, *2*, 384–393. doi: 10.11648/j.sjph.20140205.13

[CIT0010] Hazra, A., & Gogtay, N. (2016). Biostatistics series module 6: Correlation and linear regression. *Indian Journal of Dermatology*, *61*, 593. doi: 10.4103/0019-5154.193662 27904175 PMC5122272

[CIT0011] Ishizaki, A., Bouscaillou, J., Luhmann, N., Liu, S., Chua, R., Walsh, N., … Easterbrook, P. (2017). Survey of programmatic experiences and challenges in delivery of hepatitis B and C testing in low- and middle-income countries. *BMC Infectious Diseases*, *17*, 696. doi: 10.1186/s12879-017-2767-0 29143609 PMC5688462

[CIT0012] Kabir, A., Tabatabaei, S. V., Khaleghi, S., Agah, S., Faghihi Kashani, A. H., Moghimi, M., … Alavian, S. M. (2010). Knowledge, attitudes and practice of Iranian medical specialists regarding hepatitis B and C. *Hepatitis Monthly*, *10*, 176–182. 22308136 PMC3269081

[CIT0013] Kisic-Tepavcevic, D., Kanazir, M., Gazibara, T., Maric, G., Makismovic, N., Loncarevic, G., & Pekmezovic, T. (2017). Predictors of hepatitis B vaccination status in healthcare workers in Belgrade, Serbia, December 2015. *Eurosurveillance*, *22*, 30515. doi: 10.2807/1560-7917.ES.2017.22.16.30515 28449736 PMC5404481

[CIT0014] Konlan, K. D., Aarah-Bapuah, M., Kombat, J. M., & & Wuffele, G. M. (2017). TOPIC: “The level of nurses’ knowledge on occupational post exposure to hepatitis B infection in the tamale metropolis, Ghana”. *BMC Health Services Research*, *17*, 254. doi: 10.1186/s12913-017-2182-7 28381257 PMC5382446

[CIT0015] Lamberti, M., De Rosa, A., Garzillo, E. M., Corvino, A. R., Sannolo, N., De Pascalis, S., … Nienhaus, A. (2015). Vaccination against hepatitis b virus: Are Italian medical students sufficiently protected after the public vaccination programme? *Journal of Occupational Medicine and Toxicology*, *10*, 41. doi: 10.1186/s12995-015-0083-4 26539242 PMC4632277

[CIT0016] Maltezou, H. C., & Poland, G. A. (2014). Immunization of healthcare providers: A critical step toward patient safety. *Vaccine*, *32*, 4813. doi: 10.1016/j.vaccine.2014.05.046 24863487

[CIT0017] Martinez, B. A. F., Leotti, V. B., Silva, G. D. S. E., Nunes, L. N., Machado, G., & Corbellini, L. G. (2017). Odds ratio or prevalence ratio? An overview of reported statistical Methods and appropriateness of interpretations in cross-sectional studies with dichotomous outcomes in veterinary medicine. *Frontiers in Veterinary Science*, *4*, 193. doi: 10.3389/fvets.2017.00193 29177157 PMC5686058

[CIT0018] Mesfin, Y. M., & Kibret, K. T. (2013). Assessment of knowledge and practice towards hepatitis B among medical and health Science students in Haramaya University, Ethiopia. *PLOS One*, *8*, e79642. doi: 10.1371/journal.pone.0079642 24278151 PMC3836877

[CIT0019] Ministry of Health. (2018a). *National health facility master list 2018. A complete list of all health facilities in Uganda [online]*. Kampala, Uganda: Ministry of Health. Retrieved April 01, 2020, from: http://library.health.go.ug/publications/health-facility-inventory/national-health-facility-master-facility-list-2018

[CIT0020] Ministry of Health. (2018b). *Uganda commended for efforts in the fight against Hepatitis B* [Online]. Retrieved from: https://health.go.ug/content/uganda-commended-efforts-fight-against-hepatitis-b

[CIT0021] Mæstad, O., Torsvik, G., & Aakvik, A. (2010). Overworked? On the relationship between workload and health worker performance. *Journal of Health Economics*, *29*, 686–698. doi: 10.1016/j.jhealeco.2010.05.006 20633940

[CIT0022] Mursy, S. M.-E. M., & Mohamed, S. O. O. (2019). Knowledge, attitude, and practice towards hepatitis B infection among nurses and midwives in two maternity hospitals in Khartoum, Sudan. *BMC Public Health*, *19*, 1597. doi: 10.1186/s12889-019-7982-8 31783744 PMC6884767

[CIT0023] National Drug Authority. (2018). *Press statement on final investigations report on suspected falsified hepatitis B vaccine found on the Ugandan market in March* 2018 [Online]. Kampala, Uganda: National Drug Authority. Retrieved March 20, 2020, from: https://www.nda.or.ug/press-statement-on-final-investigations-report-on-suspected-falsified-hepatitis-b-vaccine-found-on-the-ugandan-market-in-march-2018/

[CIT0024] Pourhoseingholi, M. A., Baghestani, A. R., & Vahedi, M. (2012). How to control confounding effects by statistical analysis. *Gastroenterology and Hepatology from Bed to Bench*, *5*, 79. 24834204 PMC4017459

[CIT0025] Ray, G. (2017). Current scenario of hepatitis B and its treatment in India. *Journal of Clinical and Translational Hepatology*, *5*, 277. 28936409 10.14218/JCTH.2017.00024 PMC5606974

[CIT0026] Said, A., & Jou, J. H. (2014). Hepatitis B vaccination and screening awareness in primary care practitioners. *Hepatitis Research and Treatment*, *2014*, 373212. doi: 10.1155/2014/373212 24729872 PMC3963216

[CIT0027] Schillie, S. F., Murphy, T. V., Sawyer, M., Ly, K., Hughes, E., Jiles, R., … Ward, J. W. (2013). *CDC guidance for evaluating health-care personnel for hepatitis B virus protection and for administering postexposure management*. 24352112

[CIT0028] Schmidt, A. F., & Finan, C. (2018). Linear regression and the normality assumption. *Journal of Clinical Epidemiology*, *98*, 146–151. doi: 10.1016/j.jclinepi.2017.12.006 29258908

[CIT0029] Shi, Y.-H., & Shi, C.-H. (2009). Molecular characteristics and stages of chronic hepatitis B virus infection. *World Journal of Gastroenterology*, *15*, 3099–3105. doi: 10.3748/wjg.15.3099 19575488 PMC2705731

[CIT0030] Ssekamatte, T., Mukama, T., Kibira, S. P. S., Ndejjo, R., Bukenya, J. N., Kimoga, Z. P. A., … Mutyoba, J. N. (2020). Hepatitis B screening and vaccination status of healthcare providers in Wakiso district, Uganda. *PLOS One*, *15*, e0235470. doi: 10.1371/journal.pone.0235470 32645078 PMC7347299

[CIT0031] Stanaway, J. D., Flaxman, A. D., Naghavi, M., Fitzmaurice, C., Vos, T., Abubakar, I., … Cowie, B. (2016). The global burden of viral hepatitis from 1990 to 2013: Findings from the global burden of disease study 2013. *The Lancet*, *388*, 1081–1088. doi: 10.1016/S0140-6736(16)30579-7 PMC5100695 27394647

[CIT0032] Tatsilong, H. O. P., Noubiap, J. J. N., Nansseu, J. R. N., Aminde, L. N., Bigna, J. J. R., Ndze, V. N., & Moyou, R. S. (2016). Hepatitis B infection awareness, vaccine perceptions and uptake, and serological profile of a group of health care workers in Yaoundé, Cameroon. *BMC Public Health*, *16*, 706. doi: 10.1186/s12889-016-3388-z PMC4973072 27487845

[CIT0033] UBOS. (2018). *Statistical abstract*. Uganda Bureau of Statistics. Kampala, Uganda.

[CIT0034] Van Damme, P., Moiseeva, A., Marichev, I., Kervyn, A.-D., Booy, R., Kuriyakose, S., … Jacquet, J.-M. (2010). Five years follow-up following two or three doses of a hepatitis B vaccine in adolescents aged 11-15 years: A randomised controlled study. *BMC Infectious Diseases*, *10*, 357–357. doi: 10.1186/1471-2334-10-357 21171982 PMC3016379

[CIT0035] WHO. (2015). *Guidelines for the prevention care and treatment of persons with chronic hepatitis B infection*. Geneva, Switzerland: World Health Organization. 26225396

[CIT0036] WHO. (2017a). *Global hepatitis report 2017*. Geneva, Switzerland: World Health Organization.

[CIT0037] WHO. (2017b). *WHO guidelines on hepatitis B and C testing*. Geneva, Switzerland: World Health Organization.

[CIT0038] Yelland, L. N., Salter, A. B., & Ryan, P. (2011). Performance of the modified Poisson regression approach for estimating relative risks from clustered prospective data. *American Journal of Epidemiology*, *174*, 984–992. doi: 10.1093/aje/kwr183 21841157

[CIT0039] Ziraba, A. K., Bwogi, J., Namale, A., Wainaina, C. W., & Mayanja-Kizza, H. (2010). Sero-prevalence and risk factors for hepatitis B virus infection among health care workers in a tertiary hospital in Uganda. *BMC Infectious Diseases*, *10*, 191. doi: 10.1186/1471-2334-10-191 20587047 PMC2910699

